# Supplementary material for: Parallel Polarization State Generation
Source: Sci Rep. 2016 May 17;6:26019. doi: 10.1038/srep26019 (PMC4869035; doi:10.1038/srep26019)
Supplement: Supplementary Information [file srep26019-s1.pdf]

Supplementary Information

Parallel Polarization State Generation

Alan She[1*] and Federico Capasso[1*]
[1]Harvard John A. Paulson School of Engineering and Applied Sciences, Cambridge, MA 02138

*To whom correspondence should be addressed;
E-mail: ashe@seas.harvard.edu; capasso@seas.harvard.edu.

S1 Polarization mathematics

Polarization is commonly represented in two forms: the Jones vector and the Stokes parameters. The Jones vector is a complex 2-element vector, which describes completely polarized light by defining the phases and amplitudes of two orthogonal electric field components. The electrical field of a plane wave propagating in the z direction can be written as the following:

$$\mathbf{E}(z,t) = \left(E_{0x}\cos(kz - \omega t + \delta_x), E_{0y}\cos(kz - \omega t + \delta_y)\right) \qquad \text{(S1)}$$

The complex amplitude coefficients of equation (S1) are known as the Jones vector: $\left(E_{0x}e^{i\delta_x}, E_{0y}e^{i\delta_y}\right)$. The Stokes parameters, which were defined by G. Stokes in 1852 to mathematically describe polarized light, including partial polarization[1], are extremely useful today but were historically hampered by the inability to quantify optical intensity measurements. To derive the Stokes parameters, the equation of the polarization ellipse[2], $\frac{E_x^2}{E_{0x}^2} + \frac{E_y^2}{E_{0y}^2} - 2\frac{E_x}{E_{0x}}\frac{E_y}{E_{0y}}\cos\delta = \sin^2\delta$, where $\delta = \delta_y - \delta_x$, can be rearranged such that the grouped terms of $(E_{0x}^2 + E_{0y}^2)^2 - (E_{0x}^2 - E_{0y}^2)^2 - (2E_{0x}E_{0y}\cos\delta)^2 = (2E_{0x}E_{0y}\sin\delta)^2$ can be written as the following Stokes parameters:

$$\begin{aligned} S_0 &= E_{0x}^2 + E_{0y}^2, \\ S_1 &= E_{0x}^2 - E_{0y}^2, \\ S_2 &= 2E_{0x}E_{0y}\cos\delta, \text{ and} \\ S_3 &= 2E_{0x}E_{0y}\sin\delta. \end{aligned} \qquad \text{(S2)}$$

S_0 is the total intensity, and $\left(S_1, S_2, S_3\right)$ is the Stokes vector describing the SOP. The Stokes parameters simplify measurement of SOP enormously by requiring only 4 intensity measurements. A triangle inequality exists, in which the total intensity $S_0^2 \geq S_1^2 + S_2^2 + S_3^2$. The ratio of the length of the Stokes vector to the total intensity is the degree of polarization: $DOP = \frac{\sqrt{S_1^2 + S_2^2 + S_3^2}}{S_0}$. The Stokes vector that is normalized to a unit vector traces all possible SOPs on a mathematical object called the Poincaré sphere.

S2 Regions of coverage of Poincaré Sphere using degenerate SBVs

The degenerate SBVs take on any four of the six degenerate polarizations, which are the linear horizontal, linear vertical, linear +45°, linear -45°, right circular, and left circulation polarizations. We explored a system of SBVs in the four following polarizations: linear horizontal, linear vertical, linear +45°, and right circular. Coverage of the Poincaré sphere by possible SOP states using the above system is shown in Fig. 2a,c. Fig. S1 plots the Mercator projection of Fig. 2c on the Poincaré sphere. Each system is uniquely defined by the SOPs and the global phases ϕ_n of the SBVs that comprise it. In this particular system, all SBVs have ϕ=0°, except the SBV with linear +45° SOP, for which ϕ=180°.

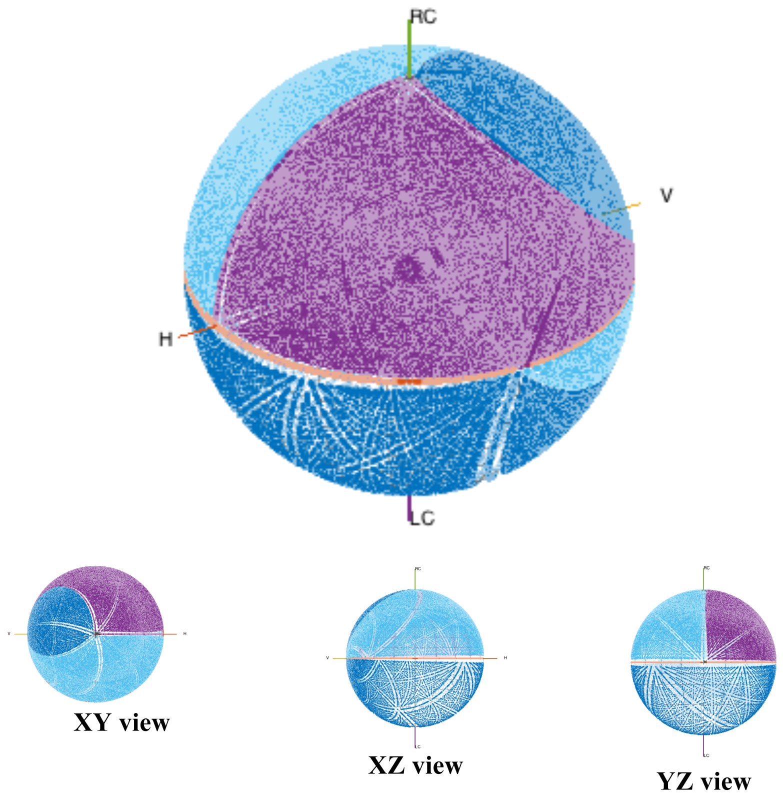

Figure S1: **Degenerate SBV coverage.** The Poincaré sphere is shown, covered by possible SOPs, as generated by linear combinations of four degenerate SBVs, in the following polarizations: linear horizontal, linear vertical, linear +45°, and right circular. All SBVs have global phases ϕ=0°, except that of the linear +45° polarization with ϕ=180°. This system is identical to that of Fig. 2a,c.

S3 Discussion of metric tensor

The uniformity of the system can be described by the metric on the Poincaré sphere, where angular separation between SOPs in Stokes space is given by $\cos\theta_{AB} = \frac{\mathbf{S}_A \cdot \mathbf{S}_B}{|\mathbf{S}_A||\mathbf{S}_B|}$ and in Jones vector space $\cos^2\frac{\theta_{AB}}{2} = \frac{|\langle E_A|E_B\rangle|^2}{\langle E_A|E_A\rangle\langle E_B|E_B\rangle}$. By describing nearby states as $E_i = C_{ij}\alpha_j$ and $E_i = C_{ij}\left(\alpha_j + \delta\alpha_j\right)$, it is possible to construct a metric tensor, where $ds^2 = d\theta^2$. For the case of coherent combination, this is the Fubini-Study metric, and for incoherent combination this is the Bures metric.

S4 Coherent versus incoherent combination

As described in the main text, coherent combination (Fig. S2) produces polarization trajectories that are sensitive to the difference in global phases ϕ_n between SBVs (Fig. S3), whereas incoherent combination necessitates a geodesic trajectory that is insensitive to ϕ_n (Fig. S4). It is possible to switch between these two combination methods to generate trajectories with degrees of coherence intermediate between the two limits by changing the mutual coherence between SBVs. Mutual coherence, or the degree of cross-correlation, can be tuned by varying the optical path length between SBVs. By having the optical length exceed the coherence length of the light source, the

SBVs no longer have a fixed phase relation and incoherently combine. Fig. S4 further illustrates the difference in polarization trajectories as generated by the two methods of combination.

The SOP measured following incoherent combination can be described as a linear sum of Stokes vectors: $\mathbf{S}_{out} = \alpha_1^2\mathbf{S}(\mathbf{C}_1) + \alpha_2^2\mathbf{S}(\mathbf{C}_2) + \alpha_3^2\mathbf{S}(\mathbf{C}_3) + \alpha_4^2\mathbf{S}(\mathbf{C}_4)$, in which $\mathbf{S}(\mathbf{C}_1)$ is the Stokes vector corresponding to the SOP of $\mathbf{C}_1$, and so on. This can be seen as the simultaneous detection of the intensities of non-interfering beams with their unaltered SOPs; hence the intensities of Stokes vectors add linearly on the detector side. The incoherent trajectory is insensitive to the relative phase difference between SBVs. Finally, the degree of polarization of the generated SOP as measured by a polarimeter has two contributors: (a) any unpolarized background originating from the unpolarized parts of the sources' signals and (b) any less than unity value of the degree of cross-correlation (mutual coherence) between combined SBVs.

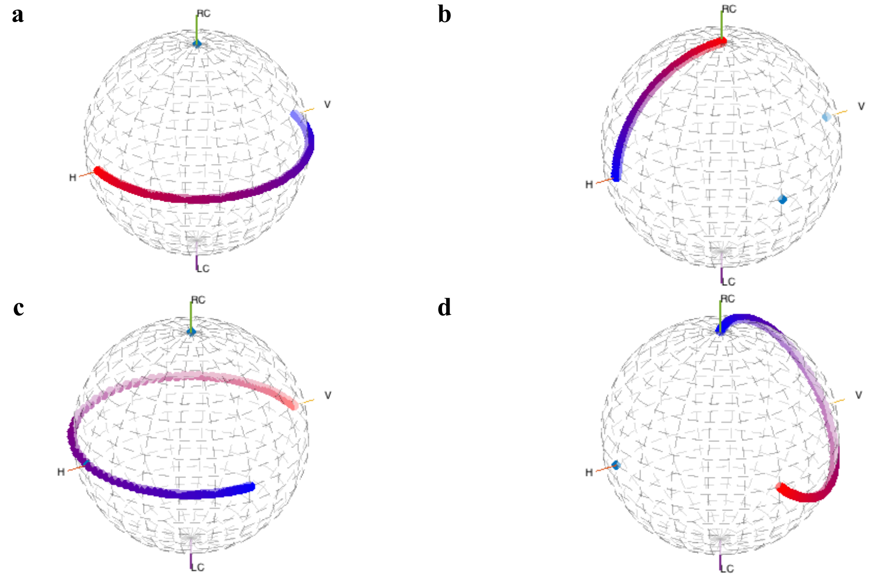

Figure S2: **Coherent polarization trajectories on the Poincaré sphere.** Trajectories are shown, where the polarization is varied from the initial state (red) to the final state (blue). Four trajectories between degenerate SOPs are plotted: **a)** linear horizontal to linear vertical, **b)** right circular to linear horizontal, **c)** linear vertical to linear +45°, and **d)** linear +45° to right circular. The global phase of the linear +45° polarization is defined to be 180° out-of-phase with respect to the other degenerate SOPs, such that in Jones vector notation it is [-1, -1].

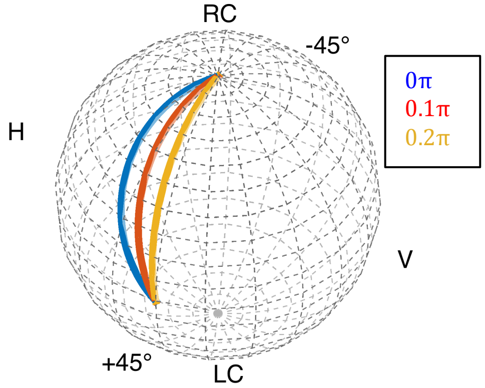

Figure S3**: Phase dependence of coherent polarization trajectories on the Poincaré sphere.** The effect on polarization trajectories by changing the relative phase between two linearly combined SOPs is shown. The polarization trajectory connecting SOPs can be modified in either direction by controlling the relative phase.

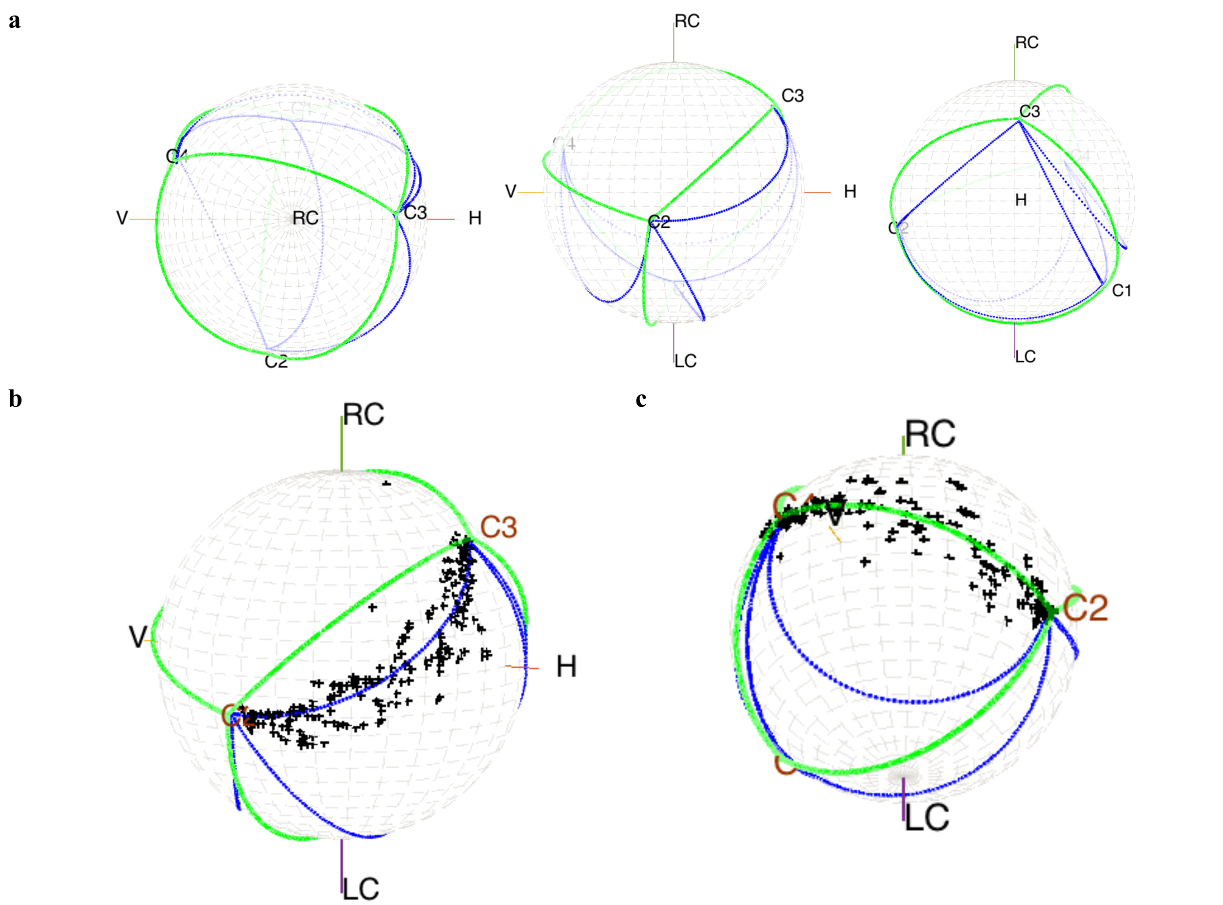

Figure S4: **Coherent and incoherent polarization trajectories on the Poincaré sphere. a)** Various perspective views of an example system. The SBVs used are labeled C1-4. Polarization trajectories are generated by coherent combination (blue line) and incoherent combination. **b)** This example system is implemented using the experimental setup described in the main text. A polarization trajectory is generated by coherent combination by keeping the optical path length between the two SBVs C2 and C3 well below the coherence length of the laser (~20 cm). The trajectory is measured by the polarimeter and the data are shown. **c)** An incoherent polarization trajectory following the geodesic path between SBVs C2 and C4 is generated by making the optical path length between SBVs much longer than the coherence length of the laser (hence reducing the mutual coherence).

S5 Comparison of the performance of our experiment with a commercial PSG

Performance characteristics of our implementation are promising, with an SOP settling time (representing speed and stability) of 3.5 μs compared to a state-of-the-art device (Thorlabs DPC5500) with 150 μs for < 10° deviation and 1 ms for < 1° deviation. However the SOP accuracy of our embodiment (5.9° error) is limited by the unstable relative phase between the four SBVs, whereas the DPC5500 can be as accurate as 0.25°. However, there is room for major improvements, in terms of both speed and accuracy, such as by using faster modulators and miniaturization; the latter would greatly increase the phase stability between SBVs and reduce the error. Realistically, we expect PSGs in the visible and telecom wavelengths, for example, to achieve the speeds of the fastest modulators available, e.g. greater than 40 GHz (lithium niobate), pushing PSG technology from the kiloradians/second regime into the gigaradians/second.

S6 Insertion loss calculation

The fundamental limitation to the efficiency of the parallel architecture stems from inefficient beam combining. The theoretical coherent beam combining efficiency for a system with N ports has been derived as the following[3]:

$$\eta = \frac{1}{N} \frac{\left| \sum_{m=1}^{N} \sqrt{P_m} \exp(j\phi_m) \right|^2}{\sum_{m=1}^{N} P_m} \tag{S3}$$

where P_m is the power and ϕ_m the phase of the mth beam. This assumes perfect alignment and coherence, so, in practice, there will be additional losses. Theoretical insertion loss (*IL*) was calculated using equation (S3), where $IL = -10\log(\eta)$, for a large number of SOPs which represented uniform coverage of the Poincaré sphere. Descriptive statistics are shown in Table S1.

A PSG constructed from a set of 4 degenerate SBVs has a greater range of insertion loss compared to that of regular tetrahedral SBVs (Fig. S5 and see standard deviation values in Table S1). This can be explained by the increased uniformity of the regular tetrahedral SBVs, which are well-separated SOPs in state space, in contrast to the degenerate SBVs, implying that on average each generable SOP will be much closer to one single SBV than the other three, leading to a large yet frequent power imbalance between the beams when combining. With the set of 4 degenerate SBVs, there is greater variability in the state space distance from each generable SOP to the SBVs.

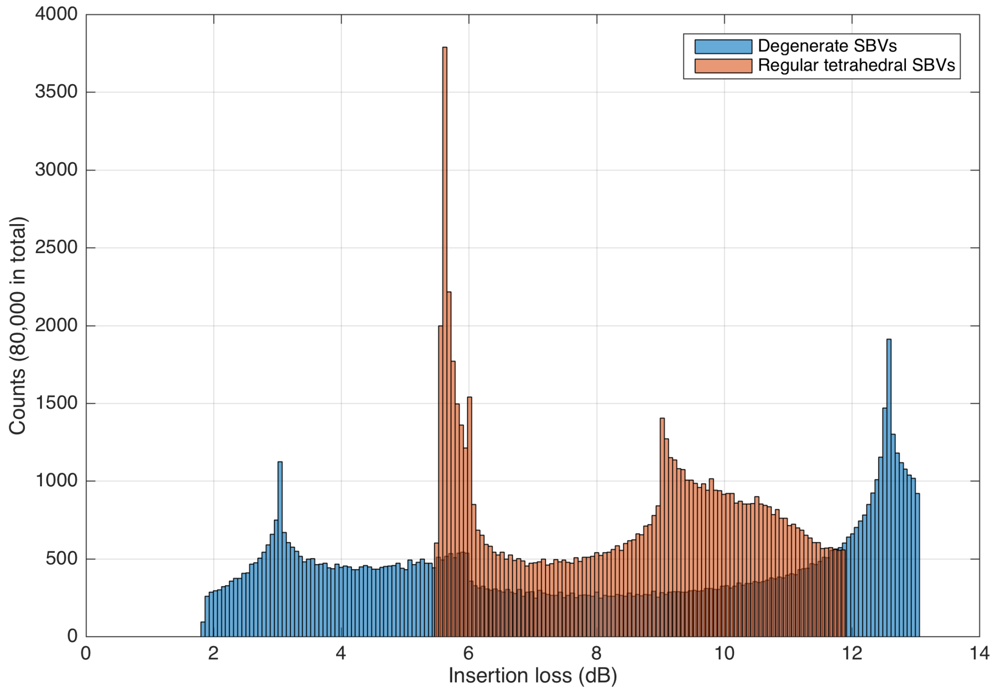

Figure S5: **Insertion loss calculation.** Insertion loss is calculated by solving equation (3) and equation (S3) for 80,000 SOPs distributed uniformly over the Poincaré sphere. The distribution of insertion losses are represented by histograms, for which a set of 4 degenerate SBVs and a set of regular tetrahedral SBVs are shown in blue and orange, respectively.

Table S1: **Insertion loss statistics for Fig. S5.**

	Degenerate SBVs			Regular tetrahedral SBVs	
	Efficiency (linear)	Insertion loss (dB)		Efficiency (linear)	Insertion loss (dB)
Mean	0.22	6.50		0.16	8.04
Minimum	0.05	13.05		0.06	11.89
Maximum	0.65	1.84		0.28	5.47
Standard deviation	0.17	4.47		0.07	2.11

References

1. Stokes, G. G. On the composition and resolution of streams of polarized light from different sources. *Trans. Cambridge Philos. Soc.* **IX,** 233–58 (1852).
2. Collett, E. *Field Guide to Polarization*. (Society of Photo Optical, 2005).
3. Link, C., Fan, T. Y. & Member, S. The Effect of Amplitude (Power) Variations on Beam Combining Efficiency for Phased Arrays. (2016). doi:10.1109/JSTQE.2008.2010232
